# Association of fat mass and obesity-associated gene rs9939609 variant with early onset obesity among bataknese and Chinese children in Indonesia

**DOI:** 10.1186/1687-9856-2015-S1-P73

**Published:** 2015-04-28

**Authors:** Siska Mayasari Lubis, Jose Batubara, Harun Alrasyid Damanik, Miswar Fattah

**Affiliations:** 1 Pediatric Endocrinology Division, Child Health Department Medical School, University of Sumatera Utara, H.Adam Malik General Hospital, Medan, Sumatera Utara, Indonesia; 2 Pediatric Endocrinology Division, Child Health Department Medical School, University of Indonesia, Cipto Mangunkusumo General Hospital, Jakarta, Indonesia; 3 Department of Nutritional Sciences, Medical School, University of Sumatera Utara, Medan, Sumatera Utara, Indonesia; 4 Department of Molecular Biology, Prodia Widyahusada Laboratory, Jakarta, Indonesia

## Background

Obesity is becoming a worldwide epidemic in modern society, it is prevalent in individuals of both genders and of all ages, socio-economic strata, and ethnic group. Several studies have reported an association between rs9939609 polymorphisms of the FTO gene and obesity. However, this association has not yet been studied among the Indonesian children.

## Aims

This is the first study in Indonesia to investigate the association between fat mass and obesity-associated gene rs9939609 variant with early onset obesity among Bataknese (n=94) and Chinese (n=66) children in Medan, North Sumatera, Indonesia.

## Methods

We conducted a case control study in ten elementary schools in Medan, North Sumatera, Indonesia. Case group (n=105) were children with early onset obesity and control group (n=55) were normal weight children. The inclusion criteria were Bataknese and Chinese children, aged 6-12 years with early onset obesity. We examined body weight and height, body mass index, waist circumference. Genotyping was performed using a TaqMan assay for rs9939609 polymorphism.

## Results

A total of 160 children between 6 and 12 years old were recruited in this study. The distribution of genotypes and alleles was significantly different among ethnicities (P=0,004), but no association was found for early onset obesity, related anthropometric measurements and gender. FTO rs9939609 allele was not associated with central obesity.

## Conclusion

Our study showed there were no associations between fat mass and obesity-associated gene rs9939609 variant with early onset obesity. However, the prevalence of genotypes for FTO rs9939609 in our study were similar with other study in China and Malaysia.

